# The effect of E-homework on K-12 students’ academic achievements: a meta-analysis study

**DOI:** 10.3389/fpsyg.2026.1758739

**Published:** 2026-01-30

**Authors:** Zhiping Yang, Yating Yu, Ruyi Chen

**Affiliations:** School of Education, Hunan University of Science and Technology, Xiangtan, Hunan, China

**Keywords:** academic achievements, assignment performance, exam performance, E-homework, K-12

## Abstract

Although E-homework has gained increasing popularity with the digital transformation of education, its actual effectiveness remains widely debated in academia. This study employed a meta-analysis to comprehensively evaluate the impact of E-homework on K-12 students’ academic achievements based on experimental research conducted between 2000 and 2025. Literature was sourced from Chinese databases (CNKI, Wanfang, etc.) and English databases (Web of Science, Science Direct, etc.), resulting in the inclusion of 26 empirical studies that met predefined criteria. In addition to the overall effect analysis, subgroup analyses were conducted. The results revealed a significant positive effect of E-homework on academic achievement, with an overall effect size of Hedges’ *g* = 0.309. Subgroup analysis further indicated that the enhancing effect of E-homework was more pronounced on assignment scores compared to exam scores. Furthermore, both subject domain and school level were found to moderate the effectiveness of E-homework.

## 1 Introduction

Homework is generally defined as written or oral tasks assigned by teachers, which require students to complete independently or collaboratively outside of class. Its purpose is to prepare for the learning of new knowledge, or to consolidate, expand, practice and refine the newly learned content (Cooper, 1989; Corno, 1996). As an important way to promote students’ after-class learning, homework has long been a hot topic of concern among educational researchers, policymakers and front-line teachers, yet there has been ongoing controversy over its effectiveness for a long time.

With the increasing popularization of educational informatization, especially against the backdrop of the profound impact of the COVID-19 pandemic on traditional teaching models (Yang et al., 2020), the application of computer technology in teaching has become more widespread, and the use of E-homework has shown a trend of rapid growth (Zhou et al., 2013). “E-homework” is also often referred to as “web-based homework” or “online homework systems.” Different from traditional paper-and-pencil homework, it usually refers to a computer-based homework system that enables automatic grading and instant feedback (Leong and Alexander, 2014). From a broader perspective, E-homework is part of e-learning, which is defined as learning and teaching activities carried out through network technology (Garrison, 2003). E-homework is defined as a computerized homework system that can operate “anytime and anywhere.” After students submit their answers via the network, the system can immediately determine whether the answers are correct or not and allow immediate corrections, without relying on in-class submission or delayed marking by teachers (Bonham et al., 2003). Currently, widely used E-homework systems include WebAssign, WeBWorK, Blackboard, MyMathLab, CalcPortal (Lucas, 2012; Callahan, 2016; Mendicino et al., 2009; Patall et al., 2008; Singh et al., 2011), as well as platforms such as ASSISTments developed by Carnegie Mellon University (Anderson et al., 1985; John and Ray, 1991).

In colleges and universities, E-homework has become very popular, and studies have proven that the learning value of E-homework is higher than that of traditional homework (from A Comparison of Traditional Homework to Computer-Supported Homework). For example, Nuri Balta and others conducted a study on the physics homework of 85 undergraduate students. The study found that Socrative, as an E-homework platform, can effectively improve students’ physics academic performance and has been positively recognized by students (from Using Socrative as an Online Homework Platform to Increase Students’ Exam Scores). In the K-12 field, Mendicino and others conducted a study on 76 students from four fifth-grade classes. The results showed that students who completed web-based homework achieved higher learning gains compared with their peers, with an effect size of 0.61(Mendicino et al., 2009).

Compared with traditional paper-and-pencil homework, E-homework has advantages in multiple aspects. It can provide students with instant feedback and support them in self-correction, thereby increasing practice opportunities and consolidating newly learned knowledge. In addition, this system can also reduce the workload of teachers in marking homework, facilitate real-time monitoring of students’ learning progress, and improve the efficiency of teaching support (Figueroa-Cañas and Sancho-Vinuesa, 2021; Magalhães et al., 2020) More importantly, E-homework has been found to stimulate students’ interest in learning. Survey results show that although traditional homework is often considered monotonous and unappealing, online homework, with its interactive interface and attractive visual effects, is more likely to arouse learners’ interest, especially in cases of high interactivity (Magalhães et al., 2020). However, at the same time, there are also some opposing views on E-homework. Studies have pointed out that many online homework systems fail to provide detailed explanations for incorrect answers; the function of multiple submissions may lead students to rely on repeated trial and error rather than in-depth thinking about problems; and the mere emphasis on the correctness of the final answer may weaken the importance of understanding the problem-solving process (Bonham et al., 2001). The convenience of accessing online information may tempt students to plagiarize or purchase answers, thereby undermining the learning objectives of homework and reducing the authenticity of the homework process (Bonham et al., 2001). Some analyses have proven that there is no significant difference in the effect of E-homework and traditional homework in improving students’ academic performance. Among them, Bonham found through an analysis of students studying calculus and basic algebra that there were no statistical differences between the E-homework group and the traditional homework group in terms of regular exams, concept tests, and experimental performance (Bonham et al., 2001). This indicates that the homework medium itself has a limited impact on learning effects. Similar conclusions have also been found in studies of other disciplines (Alghazo, 2005; Demirci, 2007; Kodippili and Senaratne, 2008; Palocsay and Stevens, 2008). Scott W. Bonham and others adopted a quasi-experimental design. By comparing the scores of the paper-and-pencil homework group and the web-based homework group in exams, homework, experiments, and concept tests, they found that the form of homework (web-based or paper-based) had no significant impact on students’ academic performance (from Comparison of Student Performance Using Web and Paper-Based Homework in College-Level Physics).

From the perspective of current research, inconsistent findings regarding the effectiveness of electronic assignments indicate that their impacts are shaped by multiple factors, including assignment release type, intervention duration, feedback form, students’ academic stage, and subject domain. Such inconsistency is not unique to electronic homework. Research in technology-enhanced education suggests that the effectiveness of digital instructional interventions is highly contingent on learner characteristics and implementation contexts rather than on the technology itself. Recent evidence published in Frontiers further highlights the role of students’ digital competencies and contextual readiness in shaping learning outcomes (Sergeeva et al., 2025). Accordingly, variations in electronic homework effects are likely attributable to contextual and moderating factors, underscoring the need for a systematic examination of when and under what conditions electronic assignments are most effective.

### 1.1 Publication type

The type of publication of the article may affect the reporting results of the effect of E-homework intervention. Existing meta-analysis indicates that formally published journal papers tend to report a higher positive effect than dissertations or conference papers, which may be related to journal publication bias, that is, a greater tendency to publish studies with statistically significant results (Cooper, 1989; Lipsey and Wilson, 2001). For instance, Kingston and Nash found in their meta-analysis of mathematical formative evaluation that the average effect size of peer-reviewed journal papers was higher than that of unpublished studies (Kingston and Nash, 2011). This difference might stem from the selective reporting of results or variations in research quality. Similar phenomena also exist in the field of educational technology. Conference papers or technical reports often present exploratory or pilot results, with small effect values and large fluctuations (Mendicino et al., 2009; Chou et al., 2017). Therefore, when interpreting the integrated research results of E-homework, it is necessary to distinguish between peer review and non-peer review sources, as the dissemination channels of the research may systematically affect the effectiveness level of the reports.

### 1.2 Intervention duration

The duration of the experiment is also an important factor causing differences in research results. Short-term studies (usually lasting less than 4 weeks) are more likely to capture the “novelty effect” of technology use, that is, students show a higher learning motivation in the early stage due to novelty, thereby improving their academic performance (Roschelle et al., 2016; Vanzo et al., 2024). For instance, Wu et al. implemented a three-week E-homework intervention in primary school mathematics classrooms in China (Wu et al., 2024). The results showed that both the homework completion rate and academic performance significantly improved. This outcome might partly reflect the positive response of students when they first came into contact with new technologies. In contrast, long-term intervention is more capable of testing the sustainable effect of e-work, but its effect value often weakens or even becomes insignificant after the novelty wears off and the implementation challenges increase (Schubert, 2012; Taylor, 2019). Trautwein pointed out that the relationship between homework and academic performance may present a “diminishing marginal benefit” pattern, that is, in the absence of adjustments to task design and feedback mechanisms, the benefits of long-term homework may tend to level off or even decline (Trautwein, 2007). Therefore, when comparing the research results of E-homework, the variable of experimental duration must be taken into account. From short-term pilot projects to year-round implementation, there may be significant differences in the results.

### 1.3 Feedback type

Hattie and Timperley defined feedback as “providing learners with information about their performance,” and emphasized that its core values lie in two points: timeliness (quick response to behavioral results) and learning components (including guidance that promotes understanding) (Hattie and Timperley, 2007). In traditional pen-and-paper homework, there is often a delay in correction and feedback (Smolira, 2008), while the immediate feedback mechanism of E-homework can effectively make up for this deficiency (Mendicino et al., 2009). Existing studies have shown that immediate feedback can not only provide students with specific information on the problem-solving process (reflecting the learning component), but also promote the improvement of their self-regulation ability through rapid error correction (Elawar and Corno, 1985; Shute, 2008).

In the field of mathematics, the research by Peter Swire et al. shows that the average effect size of instant feedback on E-homework reaches 0.301. Kingston and Nash conducted a meta-analysis of 19 studies on mathematical formative assessment and found that the average effect size was 0.17 (95% CI: 0.14–0.20) (Kingston and Nash, 2011). These data jointly verified the universal value of feedback in enhancing learning outcomes. However, the effect of feedback is not absolute, and its specific manifestations can vary significantly depending on the type and application context. For instance, the research results of Kulik indicate that in the concept processing and practice phase, the effect size of immediate feedback is 0.28, while in the test scenario, the effect size of delayed feedback is higher (0.36) (Kulik and Kulik, 1988). Similarly, Clariana et al. found that for low-difficulty tasks, immediate feedback is the most effective, while for high-difficulty tasks, delayed feedback has more advantages (Clariana et al., 2000). This indicates that the applicability of immediate feedback needs to be comprehensively judged in combination with the nature of the task and the scenario.

Furthermore, not all forms of feedback lead to significant benefits. From the perspective of feedback format, some studies point out that simple correct/incorrect feedback has limited effectiveness, and combining instant feedback with teacher guidance—such as teachers providing targeted explanations based on feedback data—is more conducive to improving academic performance (Singh et al., 2011). In terms of content characteristics, some studies have found no significant difference in the depth of feedback content—for example, between “instructional feedback with explanations” and “basic feedback that only indicates correctness” (Williams, 2012). This implies that the core value of instant feedback may depend more on “timeliness” and “interactivity” rather than mere content complexity. Additionally, from the perspective of learner characteristics, students’ existing academic levels may also moderate the effectiveness of feedback. Differences in how high-achieving and low-achieving students utilize feedback—such as the former being more skilled at independently adjusting strategies through feedback—deserve further exploration. Therefore, the instant feedback unique to E-homework does not necessarily improve academic performance. Its effectiveness is the combined result of feedback format, content characteristics, learner levels, and application scenarios, requiring more systematic analysis.

### 1.4 School level

In the research of traditional pen-and-paper homework, the school level is generally regarded as an important factor influencing the effectiveness of homework. Cooper’s classic meta-analysis indicates that the correlation coefficient between homework and academic performance of primary school students is 0, that of middle school students is 0.07, and that of high school students increases to 0.25 (Cooper et al., 2006), showing a trend of enhanced correlation as the school stage rises. In the field of E-homework, the differences in school level also significantly affect the research results. For instance, Mendicino, Heffernan and Feng found in an experiment conducted in four fifth-grade classes that the effect size of E-homework compared to pen-and-paper homework was as high as 0.61 (Heffernan and Feng, 2006), demonstrating a significant advantage. However, in the higher education stage, some studies such as Singh et al. have found that the difference in academic performance between E-homework and traditional homework is not significant, suggesting that the school level may be an important moderating factor (Singh et al., 2011).

### 1.5 Subject domain

Although only a few studies have explored whether there are differences in the effects of different types of homework (Paschal et al., 1984; Trautwein, 2007), but the significant role of subject types in homework research cannot be ignored (Lu and Huiyong, 2020). From the included literature, it can be seen that current research on E-homework is more concentrated in the discipline of mathematics. In the K12 stage, research has found that E-homework has a significant positive impact on math grades. For instance, Roschelle et al. found in a study involving 2,850 seventh-grade students that the effect size of E-homework reached 0.18 (Roschelle et al., 2016). However, some studies suggest that traditional pen-and-paper homework may lead to more positive performance in certain math tasks. In addition, the impact of E-homework on academic performance is also related to the characteristics of the question types. Some studies have pointed out that within the same subject, E-homework has a more significant improvement effect on objective questions, while its influence on open-ended questions is relatively limited (Vanzo et al., 2024; Chou et al., 2017). In the field of language learning, Vanzo et al. conducted a randomized controlled experiment in Italian high school English classrooms, using GPT-4-assisted interactive E-homework to replace traditional homework (Vanzo et al., 2024). The results showed that students’ grammar scores and learning engagement significantly improved, and a high level of student satisfaction was achieved. This indicates that the effect of E-homework is not uniform across different subjects and question types. Its mechanism of action may be closely related to the characteristics of the subjects, the types of question types, and the differences in students’ investment in different subjects (Trautwein, 2007; Lu and Huiyong, 2020).

In summary, existing studies have not reached a consistent conclusion regarding the effectiveness of electronic homework in improving students’ academic performance. Although several meta-analyses have compared traditional homework with electronic homework, systematic and quantitative investigations focusing specifically on the K-12 stage remain limited. In the present study, K-12 refers to primary, middle, and high school education, irrespective of differences in national schooling systems. This categorization follows the school level descriptions reported in the original studies, rather than imposing a new classification scheme across different national education systems. Within this scope, E-homework is operationally defined as homework activities assigned and completed through digital or web-based platforms that allow online submission and technology-mediated feedback, excluding purely offline or paper-based assignments. This definition was applied consistently throughout study screening and coding to ensure conceptual coherence across the included literature.

Moreover, the mechanisms through which key moderating variables—such as school level, subject domain, and feedback type—influence the relationship between electronic homework and academic achievement remain inconclusive. Notably, K-12 students differ substantially from university students in learning habits, technological proficiency, and self-regulation abilities. They tend to be more sensitive to feedback mechanisms, interaction formats, and task design features, making instructional media and homework design particularly influential for their learning outcomes (Mendicino et al., 2009). Therefore, conducting a systematic and comprehensive meta-analysis focusing on the K-12 population is essential not only for clarifying the overall effect of electronic homework, but also for identifying the conditions under which it is most effective. Such evidence can provide more targeted empirical support for homework design, educational informatization policies, and instructional practice in basic education.

### 1.6 Research questions

*RQ1*: What is the magnitude and statistical significance of the overall effect of E-homework on K-12 students’ academic achievements?

*RQ2*: Does the effect of E-homework differ significantly between its impact on academic achievements and its impact on exam performance?

*RQ3*: To what extent is the effect of E-homework moderated by key variables, including: publication type; intervention duration; feedback type; school level; subject domain.

## 2 Methods

### 2.1 Literature search

To ensure the timeliness and comprehensiveness of the review, the literature search was restricted to studies published between January 2000 to March 2025. The search process was conducted in two stages.

In the first stage, a systematic search was performed using predefined keywords related to electronic homework, including “E-homework,” “electronic homework,” “online homework,” “digital homework,” and “web-based homework.” These terms were applied to both English- and Chinese-language databases. The Chinese databases included CNKI, Wanfang, and *VIP,* while the English databases included Web of Science, ScienceDirect, SpringerLink, Wiley, and ProQuest. The last search was conducted on March, 2025. Eligible publication types included journal articles, conference papers, doctoral and master’s theses, and research reports. Search strings were adapted to the syntax of each database and combined with Boolean operators (e.g.*, “E-homework” OR “online homework” AND “academic achievement”*). Search terms were adapted slightly to the syntax requirements of each database while maintaining semantic consistency across all searches. In the second stage, backward citation tracking was conducted by manually screening the reference lists of all eligible studies to identify additional relevant literature. After removing duplicate records using reference management software followed by manual verification, and subsequently screening titles, abstracts, and full texts according to the inclusion criteria, 26 studies were retained for the final meta-analysis.

### 2.2 Inclusion and exclusion criteria

**Figure 1:**
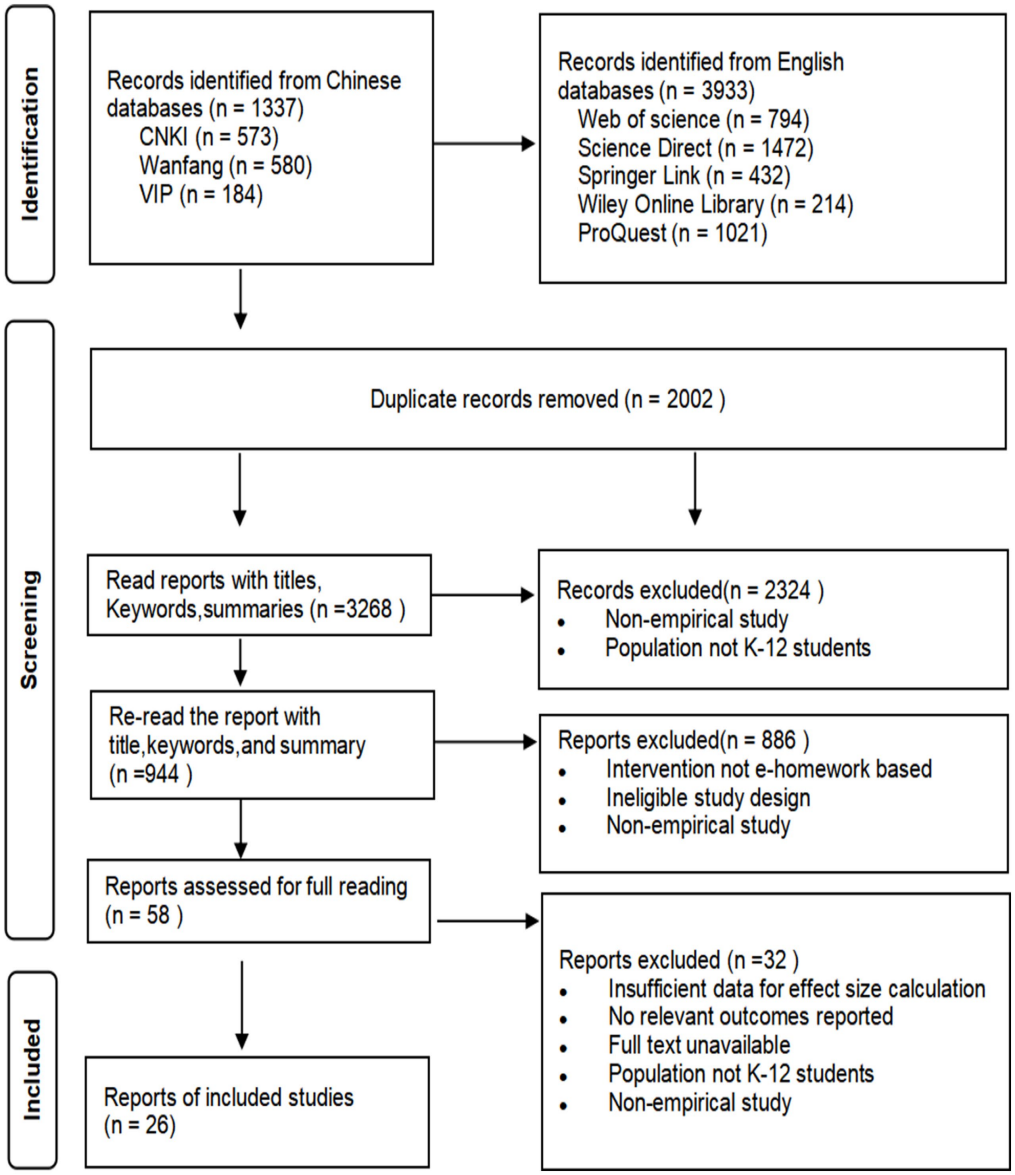
PRISMA flow diagram of study identification, screening, eligibility, and inclusion.

The study followed the Preferred Reporting Items for Systematic Reviews and Meta-Analyses (PRISMA) guidelines to determine the inclusion of studies. The detailed study selection process is illustrated in the PRISMA flow diagram (Figure 1). A study was eligible if it met all of the following seven criteria:

(1) Published between 2000 and 2025 in either Chinese or English.
(2) The target population consisted of K-12 students (i.e., elementary, middle, or high school); studies focusing on preschool or higher education were excluded.
(3) The study was an empirical primary investigation examining the effects of E-homework on K-12 students’ academic achievements; secondary analyses and literature reviews were excluded.
(4) The research design was empirical and provided sufficient statistical information (e.g., mean, standard deviation, sample size, t value, *F* value) to enable effect size computation.
(5) The study assessed at least one academic outcome, such as assignment performance or exam performance.
(6) Outcome measures could be clearly categorized as assignment performance or exam performance.
(7) The study reported sufficient information on at least one moderator variable (e.g., publication type, intervention duration, feedback type, school level, or subject domain).

Following these criteria, 26 studies were included in the meta-analysis: 3 in Chinese and 23 in English, comprising 15 journal articles, 8 dissertations, and 3 conference papers. A single effect size was extracted per study, resulting in a total of 26 independent effect sizes for synthesis.

### 2.3 Coding framework

Meta-analysis requires the systematic extraction and coding of information from all included studies, transforming descriptive information into analyzable quantitative variables. In this study, a standardized coding framework was developed based on prior meta-analytic research and refined through pilot coding.

The coding scheme comprised two major categories: (1) descriptive information, including article title, author(s), publication year, and publication source; and (2) variable information, encompassing the independent variable (E-homework), dependent variables [exam performance (EP) and assignment performance (AP)], and moderator variables (publication type, intervention duration, subject area, school level, and feedback type). Publication type was coded as journal article (J), conference paper (C), or thesis (T). school level was classified as elementary school (ES), middle school (MS), or high school (HS), based on the school level descriptions reported in the original studies (e.g., sample characteristics or institutional context), rather than being reclassified according to country-specific schooling systems. This approach was adopted to ensure consistency in moderator coding and to reduce potential bias arising from cross-national differences in educational structures. Subject area was coded as mathematics (Math), language-related subjects (Lang), or other subjects (Oth; e.g., physics, history). Feedback type was categorized as instructional feedback (Ins) or basic feedback (Bas). Intervention duration was classified as short-term (ST; 1–4 weeks), medium-term (MT; 5–12 weeks), or long-term (LT; 13 weeks or more).

All studies were independently coded by two trained coders using a standardized coding protocol. Inter-coder agreement reached 95%, and any discrepancies were resolved through discussion and consensus, with a third researcher experienced in meta-analytic procedures serving as an adjudicator when necessary.

To avoid statistical dependency among multiple effect sizes within the same study, this meta-analysis followed the principle of “one effect size per study.” When a study reported multiple outcome measures or comparisons, we applied the following predefined hierarchy to select the most representative effect size:

(1) Primary Principle: Prioritize the effect size for the primary outcome measure specified by the authors of the study.
(2) If no primary outcome is specified:

① Among multiple related measures (e.g., multiple subtests of math scores), we selected the most comprehensive or overall score that best represents the core goal of the intervention.
② Among different types of measures, we selected the objective or behavioral measure that most directly reflects the intervention’s core mechanism.
③ Among measurements at different time points, we selected the final follow-up measure to assess the sustained effects of the intervention.

(3) For multi-group comparison studies (e.g., multiple intervention groups vs. a control group), we selected the comparison between the intervention group most aligned with the intervention theory and the control group.

Although multilevel models can address the issue of effect size dependency, the primary goal of this study was to estimate the overall effect across studies, and some subgroups had a relatively small number of studies, making multilevel modeling less suitable as it could reduce the robustness of the estimates. By aggregating a single effect size per study, we aimed to minimize potential biases arising from hierarchical complexity and statistical dependencies. At the same time, we maintained the reliability of our results through sensitivity analysis and transparent reporting rules.

### 2.4 Data analysis

This study employed Comprehensive Meta-Analysis (CMA) version 3.0 to compute effect sizes. The standardized mean difference (Hedges’ g) was adopted as the primary effect size index, with small-sample bias correction applied. Between-study heterogeneity was assessed using the Q statistic and the I^2^ index. Given the expected variability across studies, a random-effects model was utilized. To examine the potential influence of moderators, both moderator analyses and subgroup analyses were conducted, focusing on publication type, intervention duration, feedback type, school level, and subject area. Publication bias was evaluated using funnel plots, Egger’s regression test, and the trim-and-fill method.

## 3 Results

### 3.1 Publication bias test

**Figure 2:**
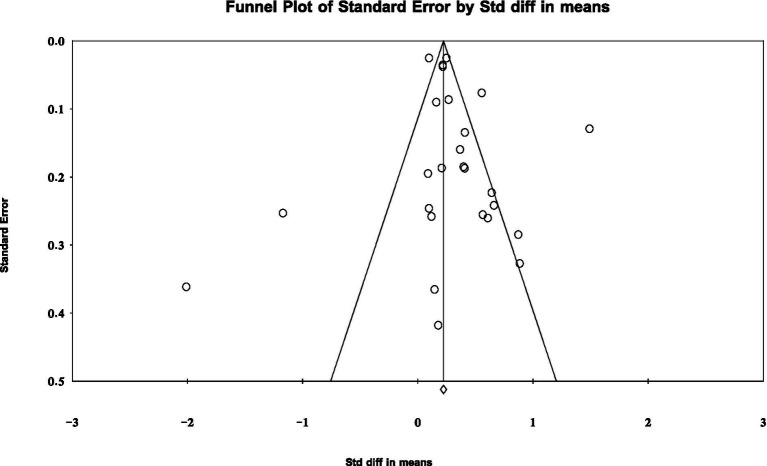
Funnel plot for assessing publication bias.

Publication bias refers to the systematic deviation caused by the fact that published literature does not fully reflect the actual research results (Dickersin and Min, 1993). To reduce the impact of potential bias and improve the reliability and validity of the research results, we tested for publication bias before conducting the meta-analysis. Funnel plots and Egger’s regression test are commonly used detection methods (Hedges et al., 2009). This study employed both methods and used CMA V3 software to generate the funnel plot (Figure 2). The results showed that the research samples were mainly concentrated in the upper and middle parts of the funnel plot and were symmetrically distributed on both sides of the average effect value line. Only a few studies were scattered at the bottom, which usually correspond to studies with low or negative effect sizes. This result indicates that the possible missing small-sample studies have a limited impact on the overall effect size. To further verify this conclusion, we conducted an Egger’s regression test, which assesses the risk of bias by testing the significance of the regression intercept (Song and Gilbody, 1998). The test results showed that the intercept was 0.824, *t* = 1.010, df = 24, *p* = 0.323 (> 0.05), indicating no significant publication bias was found. In conclusion, the possibility that the meta-analysis results of this study are affected by unpublished or missing studies is low.

### 3.2 Heterogeneity test

In meta-analysis, assessing heterogeneity is crucial for determining whether the variation in effect sizes is due to random sampling error or differences in study characteristics (Higgins et al., 2003). This study employed Cochran’s Q statistic and the I^2^ index to test for heterogeneity. The results indicated significant differences among the included studies (*Q* = 234.492, df = 25, *p* < 0.001), with an I^2^ value of 89.34%, which is considered a high level. This suggests that the variation in effect sizes is not only due to random error but may also be influenced by contextual factors such as school level, subject, feedback form, and implementation period. Based on this, a random-effects model was used for the pooling of effect sizes in this study to obtain more robust and generalizable results.

### 3.3 Overall effect of e-homework

The overall analysis results indicate that E-homework intervention has a significant positive impact on the academic achievements of K-12 students. To control for the observed heterogeneity, this study employed a random-effects model to estimate the effect size, obtaining an overall effect size of Hedges’ *g* = 0.309 (95% CI [0.199, 0.419], *Z* = 5.513, *p* < 0.001) (Table 1). This finding suggests that students who participated in E-homework performed better academically on the whole than those who did not, verifying the effectiveness of E-homework as a teaching strategy in various educational contexts.

**Table 1:** Overall summary of meta-analytic results for the main effect.

**Model**	**k**	***g* [95% CI]**	***Z***	***p***	**Heterogeneity Statistics**		
					***Q***	***I***^***2***^ ***(%)***	***p***
Fixed Effect	26	0.223 [0.197, 0.250]	16.392	<0.001	234.49	89.34	< 0.001
Random Effects	26	0.309 [0.199, 0.419]	5.513	<0.001			
Total Between-Study Variance					τ^2^ = 0.049, τ = 0.221		

k, number of studies; g, Hedges’ g (standardized mean difference); CI, confidence interval.

### 3.4 Effects on homework and exam performance

As previously known, E-homework has a positive effect on the academic achievements of K-12 students. To further explore the specific impact of E-homework on different types of academic achievements of students, this study continues to divide academic achievements into two dimensions: “homework performance” and “exam performance,” and conducts subgroup meta-analyses, respectively. The results show that E-homework has a moderate and highly significant positive effect on students’ assignment performance (*g* = 0.368, SE = 0.057, 95% CI [0.257, 0.479], *z* = 6.514, *p* < 0.001), with almost no heterogeneity among studies (*Q* = 8.423, df = 9, *p* = 0.492, *I*^2^ = 0.000), indicating that E-homework has a stable and consistent promoting effect on students’ assignment performance. In contrast, although the effect of E-homework on students’ exam performance is also significant (*g* = 0.253, SE = 0.070, 95% CI [0.116, 0.390], *z* = 3.612, *p* < 0.001), the effect size is relatively low, and the heterogeneity is significant (*Q* = 219.100, df = 15, *p* < 0.001, *I*^2^ = 93.154), suggesting that this effect may be influenced by potential moderating factors such as sample differences or task types (Table 2). Overall, the role of E-homework in improving students’ assignment performance is more prominent and consistent. In addition, the assessment of publication bias for the two subgroups of assignment performance and exam performance shows that the funnel plot distribution is roughly symmetrical, and the Egger regression test also does not show statistical significance (homework performance: *t* = 1.7989, *p* = 0.10974), further supporting the stability and credibility of the research results.

**Table 2:** Subgroup analysis by assessment type.

Assessment type	Model	k	*g* [95% CI]	*Z*	*p*	*Q*	*I^2^* (%)
Assignment	Fixed effect	10	0.368 [0.257, 0.479]	6.514	< 0.001	8.423	0
	Random effects	10	**0.368 [0.257, 0.479]**	6.514	< 0.001		
Exam	Fixed effect	16	0.214 [0.187, 0.242]	15.272	< 0.001	219.1	93.154
	Random effects	16	**0.253 [0.116, 0.390]**	3.612	< 0.001		
Test for subgroup differences			χ^2^ = 1.710, df = 1, *p* = 0.191				

k, number of studies; g, Hedges’ g; CI, confidence interval. The test for subgroup differences is not statistically significant (*p* > 0.05). Bold values denote statistically significant pooled effect sizes at the 0.05 level.

### 3.5 Moderator effects

#### 3.5.1 Publication type

Given that the publication channels of research results may differ in terms of research quality control (such as review standards) and sample selection, this study classified the included literature into three categories: journal articles, conference papers, and dissertations, to examine their potential impact on the effect size. The results of the moderation effect analysis indicated that publication type did not significantly moderate the intervention effect of E-homework (*Q* = 1.03, *p* = 0.596). The differences in effect sizes among the three types of literature did not reach statistical significance (conference papers SMD = 0.373, 95% CI [0.015, 0.732]; journal articles SMD = 0.338, 95% CI [−0.027, 0.703]; dissertations SMD = 0.200, 95% CI [−0.231, 0.631]) (Table 3). Taken together, these findings suggest that, within the scope of the current dataset, publication type did not exhibit a clear moderating effect on the effectiveness of E-homework, although this conclusion should be interpreted with caution given the limited number of studies in each subgroup.

**Table 3:** Subgroup analysis of effect sizes by publication type.

Publication type	k	SMD (95% CI)	Weight, %	95% CI	95% CI	I^2^, 100%
				Lower	Upper	
Conference paper (Ref)	3	0.373	11.5	0.015	0.732	89.90%
Journal article	15	0.338	57.7	−0.027	0.703	
Dissertation	8	0.2	30.8	−0.231	0.631	

Test for subgroup differences (Between-group heterogeneity): χ^2^ = 1.03, df = 2, *p* = 0.596. k, number of studies; SMD, Standardized Mean Difference; CI, Confidence Interval; I2, measure of heterogeneity; Ref, Reference group.

#### 3.5.2 Intervention duration

Considering that the length of the intervention period may affect the manifestation and maintenance of the effect of E-homework, in the coding process, we divided the experimental periods into three gradients based on the experimental period situations in existing studies: short-term (1–4 weeks), medium-term (5–12 weeks), and long-term (13 weeks or more), and examined the impact of different experimental periods on students’ academic achievements. The analysis results showed that the experiment duration did not significantly moderate the intervention effect (*Q* = 1.26, *p* = 0.739). The combined effect size for long-term studies was 0.183 (95% CI [−0.109, 0.475]), for medium-term studies was 0.394 (95% CI [−0.086, 0.874]), and for short-term studies was 0.291 (95% CI [−0.071, 0.653]), and the differences were not statistically significant (Table 4). Taken together, these results suggest that no clear moderating pattern of intervention duration was detected within the current dataset. However, given the limited number of studies in each duration category, these findings should be interpreted as preliminary rather than conclusive.

**Table 4:** Subgroup analysis of effect sizes by intervention duration.

Intervention duration	k	SMD (95% CI)	Weight, %	95% CI	95% CI	I^2^, 100%
				Lower	Upper	
Long-term (Ref)	5	0.183	19.2	−0.109	0.475	88.16%
Medium-term	6	0.394	23.1	−0.086	0.874	
Short-term	13	0.291	50	−0.071	0.653	
Other	2	0.403	7.7	−0.421	1.227	

Test for subgroup differences (Between-group heterogeneity): χ^2^ = 1.26, df = 3, *p* = 0.739. k, number of studies; SMD, Standardized Mean Difference; CI, Confidence Interval; I2, measure of heterogeneity; Ref, Reference group.

#### 3.5.3 Feedback type

Feedback is an important component of E-homework, and different types of feedback may affect students’ learning motivation and strategy application. In this study, feedback types were classified based on the content of feedback into Basic Feedback (providing only result determination) and Guided Feedback (including strategic suggestions and detailed explanations) to examine the impact of feedback depth on the intervention effect. The results showed that feedback type did not significantly moderate the intervention effect (*Q* = 2.43, *p* = 0.119). The effect size for basic feedback was 0.456 (95% CI [0.242, 0.670]), and for guided feedback was 0.258 (95% CI [−0.188, 0.704]), with no significant difference (Table 5). These results suggest that, within the current dataset, no clear moderating effect of feedback type was detected. However, given the limited number of studies and variability in feedback implementation, this finding should be interpreted with caution and does not preclude the potential influence of feedback characteristics under different instructional conditions.

**Table 5:** Subgroup analysis of effect sizes by feedback type.

Feedback type	k	SMD (95% CI)	Weight, %	95% CI	95% CI	I^2^, 100%
				Lower	Upper	
Basic feedback (Ref)	7	0.456	26.9	0.242	0.67	89.30%
Guided feedback	19	0.258	73.1	−0.188	0.704	

Test for subgroup differences (Between-group heterogeneity): χ^2^ = 2.43, df = 1, *p* = 0.119. k, number of studies; SMD, Standardized Mean Difference; CI, Confidence Interval; I2, measure of heterogeneity; Ref, Reference group.

#### 3.5.4 School level

Significant differences exist in students’ cognitive levels, autonomous learning abilities, and information technology application capabilities across different academic stages, which may affect the learning effectiveness of E-homework. Based on this, this study divided the sample’s school level into elementary school, middle school, and high school to explore differences in the effectiveness of E-homework for students at different developmental stages. The results showed that academic stage had a significant moderating effect on intervention effectiveness (*Q* = 8.71, *p* = 0.0129). High school students had the highest effect size (SMD = 0.808, 95% CI [0.424, 1.192]), followed by elementary school students (SMD = 0.362, 95% CI [−0.086, 0.810]), and middle school students had the lowest (SMD = 0.194, 95% CI [−0.191, 0.579]) (Table 6). These findings suggest a potential association between school level and the effectiveness of E-homework. However, given the limited number of studies within each subgroup and the overlap of confidence intervals, this moderating pattern should be interpreted as preliminary. The observed differences may reflect developmental or contextual factors, but further evidence is needed before drawing definitive conclusions about stage-specific advantages. To enhance transparency, a forest plot illustrating the effect sizes across different school-level subgroups is provided in Supplementary Figure S1.

**Table 6:** Subgroup analysis of effect sizes by school level.

School level	k	SMD (95% CI)	Weight, %	95% CI	95% CI	I^2^, 100%
				Lower	Upper	
High school (Ref)	3	0.808	7.7	0.424	1.192	89.50%
Middle school	16	0.194	61.5	−0.191	0.579	
Primary school	8	0.362	30.8	−0.086	0.81	

Test for subgroup differences (Between-group heterogeneity): χ^2^ = 8.71, df = 2, *p* = 0.013. k, number of studies; SMD, Standardized Mean Difference; CI, Confidence Interval; I2, measure of heterogeneity; Ref, Reference group.

#### 3.5.5 Subject domain

Differences in knowledge structure and task characteristics across subject domains may influence the design and effectiveness of E-homework. Accordingly, subject area was classified into mathematics, language-related subjects, and other subjects (e.g., physics, history) to examine potential differences in intervention effects. The moderator analysis indicated that subject domain was statistically significant (*Q* = 17.68, *p* = 0.0001). The pooled effect size was largest for other subjects (e.g., physics, history) (SMD = 1.021, 95% CI [0.264, 1.778]), followed by mathematics (SMD = 0.277, 95% CI [0.114, 0.440]), while the effect size for language subjects was smaller and not statistically significant (SMD = 0.046, 95% CI [−0.250, 0.341]) (Table 7). These findings suggest that the effectiveness of E-homework may vary across subject domains. However, given the limited number of studies within certain subject categories and the relatively wide confidence intervals, these results should be interpreted with caution. The observed differences may reflect variations in task structure, assessment formats, or implementation contexts, rather than inherent subject-specific advantages or limitations. The corresponding forest plot for subject-domain subgroups is presented in Supplementary Figure S2.

**Table 7:** Subgroup analysis of effect sizes by subject domain.

Subject domain	k	SMD (95% CI)	Weight, %	95% CI	95% CI	I^2^, 100%
				Lower	Upper	
Language (Ref)	4	0.046	15.4	−0.25	0.341	85.30%
Mathematics	20	0.277	76.9	0.114	0.44	
Other	2	1.021	7.7	0.264	1.778	

Test for subgroup differences (Between-group heterogeneity): χ^2^ = 17.68, df = 2, *p* < 0.001. k, number of studies; SMD, Standardized Mean Difference; CI, Confidence Interval; I^2^, measure of heterogeneity; Ref, Reference group.

## 4 Discussion

### 4.1 The effect of E-homework on K-12 students’ academic achievements

The meta-analysis results of this study show that E-homework has a significant positive impact on the academic achievements of K-12 students (Hedges’ *g* = 0.309, *p* < 0.001), indicating that compared with traditional homework, E-homework is more conducive to the improvement of students’ academic achievements on the whole. This conclusion is consistent with the results of several international studies. For instance, Vanzo et al. found in their research on E-homework tutoring that E-homework platforms significantly enhanced students’ learning engagement and academic achievements (Vanzo et al., 2024). Feng et al. also demonstrated in their long-term follow-up study that structured E-homework support can continuously enhance students’ mathematical performance (Feng et al., 2023).

The positive effects of E-homework may stem from the following aspects: Firstly, the integration of multimodal learning resources (including text, audio, video and interactive question types) helps present knowledge in a multi-sensory way, promoting students’ understanding and memory of concepts. Secondly, E-homework platforms usually have the functions of visualizing learning progress and interaction, which can help students clearly grasp the learning pace and enhance their learning planning and self-monitoring abilities. Thirdly, the interactive design of E-homework (such as real-time quizzes, situational simulations, and gamified elements) can enhance students’ interest in learning and their participation, thereby deepening the internalization of knowledge.

### 4.2 E-homework has different effects on the K-12 students’ academic achievements under the different moderating variables

#### 4.2.1 Publication type

The results indicate that publication type (journal articles, conference papers, and dissertations) did not emerge as a statistically significant moderator of the effect of E-homework on students’ academic achievements. One possible explanation is that, across different publication formats, many studies implemented similar core features of E-homework interventions, such as online assignment platforms, automated feedback mechanisms, and progress tracking systems (Heffernan and Feng, 2006). As a result, differences in reported effect sizes may be more closely related to variations in task design, instructional integration, or implementation context rather than publication format itself. In addition, previous research in educational technology suggests that dissertations and journal articles may employ comparable research designs and data sources. However, given the limited number of studies within each publication category, these interpretations remain tentative and should be viewed as exploratory rather than definitive.

#### 4.2.2 Intervention duration

The results indicate that intervention duration did not emerge as a statistically significant moderator of the E-homework effect. One possible explanation is that core features of E-homework—such as immediate feedback, personalized practice, and learning progress tracking—may begin to function within a relatively short period of time (Vanzo et al., 2024). In several included studies, students appeared to become familiar with the platform and establish routine usage patterns within the initial weeks of implementation. As a result, extending the duration of the intervention may not necessarily lead to proportionally larger learning gains.

In addition, some long-term interventions have reported declining engagement over time, a phenomenon often described as intervention fatigue, in which students’ initial participation gradually decreases (Feng et al., 2023). Such patterns may partially offset potential advantages associated with prolonged exposure. Recent studies have also highlighted the role of digital literacy in technology-supported learning environments, suggesting that students’ ability to effectively use digital tools may influence learning outcomes (Chen, 2025). However, given the limited number of long-term studies and the absence of direct measures of engagement or digital literacy in the included data, these explanations remain speculative. Overall, the present findings suggest that no clear moderating effect of intervention duration was detected, and further research is needed to examine how short-term engagement and long-term participation jointly shape the effectiveness of E-homework.

#### 4.2.3 Feedback type

The results indicate that feedback type did not emerge as a statistically significant moderator of the E-homework effect. Several plausible explanations may account for this finding. First, within E-homework contexts, the timeliness and interactivity of feedback may play a more prominent role in supporting learning than the depth or elaboration of feedback content (Vanzo et al., 2024). Even basic feedback that promptly indicates correctness may enable immediate error correction and practice consolidation, potentially reducing observable differences between feedback types.

Second, the effectiveness of more elaborated or guided feedback may depend on learners’ engagement and self-regulatory processes (Fyfe, 2016). If students do not actively process or utilize the additional explanatory information, the potential cognitive benefits of guided feedback may not be fully realized. This possibility may be particularly relevant when tasks are relatively simple or repetitive, though such learner-level processes were not directly measured in the included studies.

Third, there was substantial variability in how guided feedback was designed and implemented across studies. In some cases, guided feedback consisted of general or non-personalized explanations, which may not have been closely aligned with students’ specific errors or learning needs, potentially limiting its effectiveness (Tran and Nguyen Thanh, 2023). Given these considerations, the present findings should be interpreted with caution. They suggest that no clear moderating effect of feedback type was detected in the current dataset, and that factors such as feedback quality, alignment, and learner engagement may warrant closer examination in future research.

#### 4.2.4 School level

The results indicate that the effect of E-homework differs across school level, with a relatively larger effect observed at the high school level. This pattern may be associated with developmental differences in learning processes across stages. Prior research suggests that older students generally demonstrate more advanced metacognitive awareness and self-regulated learning strategies (Ramdass and Zimmerman, 2011), which may enable them to make more effective use of features commonly embedded in E-homework systems, such as immediate feedback and error review (Feng et al., 2023). In contrast, younger students at the elementary and middle school levels may rely more heavily on external guidance and instructional support to fully benefit from technology-mediated homework, although such learner-level mechanisms were not directly examined in the included studies. Additionally, differences in curricular structure across school level may also play a role. High school curricula tend to be more systematically organized, which may align more closely with the structured practice formats typically provided by E-homework platforms.

Taken together, these considerations offer possible interpretations of the observed moderating effect of school level. However, given the limited number of studies within some subgroups, these findings should be interpreted cautiously and regarded as indicative rather than conclusive.

#### 4.2.5 Subject domain

E-homework appears to show relatively larger effects in subjects with more structured knowledge systems, such as mathematics and science. Learning tasks in these subjects often emphasize step-by-step problem solving, immediate feedback, and repeated practice, which may align well with common design features of E-homework platforms, including error correction and procedural reinforcement mechanisms (Tokac et al., 2019). Under such conditions, students may be better able to utilize the feedback and practice opportunities provided by E-homework to support academic performance.

In contrast, language-related disciplines tend to place greater emphasis on contextual comprehension, creative expression, and diverse communicative abilities, which are less easily operationalized through automated scoring and standardized feedback systems (Metcalfe et al., 2009). Although E-homework can still provide additional practice opportunities in language learning contexts, its instructional affordances may not fully capture the complexity of language-related learning objectives. As a result, the observed effects in language subjects appear smaller and less stable. These subject-related differences should be interpreted with caution, particularly given the limited number of studies and the heterogeneity of task designs across domains.

## 5 Suggestion

### 5.1 Implications for educational practice

The results of this study indicate that the implementation of E-homework needs to be precise and differentiated to avoid a one-size-fits-all approach. Educators should give priority to systematically integrating E-homework in highly structured subjects such as high school mathematics and science to maximize its benefits. For the elementary school stage and language subjects, emphasis should be placed on developing interactive task designs that can promote contextualized understanding and creative expression, going beyond simple answering exercises. Furthermore, research has found that E-homework significantly improves academic achievements (formative assessment). Compared to exam performance (summative assessment), suggesting that its core function should be positioned as a tool for daily learning support and process feedback rather than an exam-oriented training approach.

Although the main effect of the feedback type is not significant, the quality, timeliness and pertinence of the feedback are far more important than its form. Platform developers and teachers should collaborate to design an intelligent feedback system, with the focus on shifting from “providing feedback” to “designing feedback,” offering guiding feedback that includes step-by-step prompts, strategic suggestions, and personalized explanations to help students identify and overcome thinking barriers, rather than merely informing them of right and wrong. At the same time, it is necessary to cultivate students’ ability to self-regulate their learning by using feedback information, especially in school level such as middle school where the effect is not significant.

E-homework has the most significant effect in high school students with strong autonomous learning abilities. When promoting to lower grades, it is necessary to provide clear usage guidelines, meta cognitive strategy training, and appropriate external supervision mechanisms (such as teacher or parent reminders, learning plan templates) to help students, especially middle school students, develop the habit and confidence to effectively use digital tools.

### 5.2 Implications for educational policy and leadership

Education decision-makers should ensure that all schools, especially those in resource-poor areas, have stable network access, digital terminals and high-quality operation platforms, to guarantee the fair implementation of E-homework at the hardware level and prevent the “digital divide” from widening the academic gap.

The key to the successful integration of E-homework in education lies in teachers. Policies should support the implementation of continuous teacher professional development projects. The focus of training should not be limited to technical operations but should also cover how to design high-quality E-homework, interpret learning analysis data for precise teaching intervention, and how to organically integrate E-homework into the overall curriculum and evaluation system.

### 5.3 Implications for future research

Future research should go beyond the verification of “effectiveness” and delve deeply into revealing “why and how electronic operations take effect.” The focus is on examining the interaction between its specific design features (such as adaptive sequences, gamification mechanisms, and the accuracy of feedback) and students’ traits (such as learning motivation, cognitive styles, and meta cognitive abilities) to construct a more precise theoretical explanation model.

It is suggested that future research incorporate non-cognitive outcomes such as learning engagement, self-efficacy, collaboration ability, and digital literacy into the assessment framework to comprehensively examine the educational value of E-homework. At the same time, a “longitudinal tracking design” should be adopted to test the long-term sustainability of its effect and its potential impact on higher-order thinking skills.

Encourage researchers to adopt methods such as “design research and action research,” and collaborate with front-line teachers to develop, iterate and evaluate the implementation models of E-homework in different subjects and school stages in real classroom scenarios, with a focus on factors such as teacher support strategies and school-based implementation frameworks, so as to produce research results that have both high ecological validity and practical guiding significance.

## 6 Conclusion

### 6.1 Key finding

This meta-analysis provides quantitative evidence for the impact of E-homework on the academic achievements of students in the K-12 stage. The results indicate that E-homework has a positive effect on students’ academic achievements (Hedges’ *g* = 0.309), suggesting its effectiveness as a teaching tool. A key finding is that the impact of electronic assignments on formative assessment is more pronounced and stable (*g* = 0.368, *I*^2^ = 0.000) compared to its relatively weaker and more variable impact on summative assessment (*g* = 0.253, *I*^2^ = 93.154). This suggests that E-homework may be more effective as a learning and practice tool, rather than solely as a preparatory tool for exams. The moderating effect analysis revealed that publication type, experiment duration, and feedback type did not significantly influence the intervention effect, which implies that E-homework tends to be robust across different research designs and implementation conditions. However, significant moderating effects were observed between school level and subject domain: high school students appear to benefit the most, followed by elementary school students, with middle school students benefiting the least. In terms of academic subjects, the intervention effects were more pronounced in mathematics and other non-language subjects (e.g., physics, history), compared to language subjects. These findings provide valuable insights for the design and contextual application of E-homework.

### 6.2 Limitations

Several limitations need to be considered when interpreting these results. First, the uneven distribution of samples across different categories of moderating variables may affect the accuracy of the estimation of some effect sizes. Second, although the publication bias test results indicate a low risk, the potential impact of unpublished studies cannot be completely ruled out. Third, this study did not explore the specific design features of E-homework (such as interactivity, personalization, and the timeliness of feedback) and their mechanisms of action, which limits its practical guidance for instructional design. Finally, most of the included studies focused on short-term academic outcomes, which restricts the understanding of the long-term impact of E-homework.

### 6.3 Future research directions

Future research should further expand the evidence base by incorporating more empirical studies from different disciplines, school level and regions to enhance the generalizability of the conclusions. Meanwhile, it is necessary to delve into the mechanism by which E-homework affects learning, with a focus on the interaction between the design of E-homework and students’ characteristics. Additionally, longitudinal studies should be conducted to examine the long-term impact of E-homework on students’ higher-order thinking, learning motivation and autonomous learning ability. Addressing these issues will provide more detailed and actionable evidence to support educational technology innovation and teaching reform.

## Data Availability

The datasets presented in this study can be found in online repositories. The names of the repository/repositories and accession number(s) can be found in the article/Supplementary material.

## References

[ref1] Alghazo A. M. (2005). Comparing effectiveness of online and traditional teaching using students’ final grades. Online J. Workf. Educ. Dev. 1:6.

[ref2] Anderson J. R. Boyle C. F. Reiser B. J. (1985). Intelligent tutoring systems. Science 228, 456–462. doi: 10.1126/science.228.4698.456, 17746875

[ref3] Bonham S. Beichner R. Deardorff D. (2001). Online homework: does it make a difference? Phys. Teach. 39, 293–296. doi: 10.1119/1.1375468

[ref4] Bonham S. W. Deardorff D. L. Beichner R. J. (2003). A comparison of student performance using web and paper- based homework in college-level physics. J. Res. Sci. Teach. 40, 1050–1071. doi: 10.1002/tea.10065

[ref5] Callahan J. T. (2016). Assessing online homework in first-semester calculus. Primus 26, 545–556. doi: 10.1080/10511970.2015.1128501

[ref6] Chen F. (2025). The relationship between digital literacy and college students’ academic achievement: the chain mediating role of learning adaptation and online self-regulated learning. Front. Psychol. 16:1590649. doi: 10.3389/fpsyg.2025.1590649, 40678434 PMC12269795

[ref7] Chou P.-N. Chang C.-C. Lin C.-H. (2017). BYOD or not: a comparison of two assessment strategies for student learning. Comput. Human Behav. 74, 63–71. doi: 10.1016/j.chb.2017.04.024

[ref8] Clariana R. B. Wagner D. Roher Murphy L. C. (2000). Applying a connectionist description of feedback timing. Educ. Technol. Res. Dev. 48, 5–22. doi: 10.1007/BF02319855

[ref9] Cooper H. (1989). Synthesis of research on homework. Educ. Leadership 47, 85–91.

[ref10] Cooper H. Robinson J. C. Patall E. A. (2006). Does homework improve academic achievement? A synthesis of research, 1987–2003. Rev. Educ. Res. 76, 1–62. doi: 10.3102/00346543076001001

[ref11] Corno L. (1996). Homework is a complicated thing. Educ. Res. 25, 27–30. doi: 10.3102/0013189x025008027

[ref12] Demirci N. (2007). University students’ perceptions of web-based vs. paper-based homework in a general physics course. Eurasia J. Math. Sci. Technol. Educ. 3, 29–34. doi: 10.12973/ejmste/75371

[ref13] Dickersin K. Min Y. (1993). Publication bias: the problem that won’t go away. Ann. N. Y. Acad. Sci. 703, 135–148. doi: 10.1111/j.1749-6632.1993.tb26343.x, 8192291

[ref14] Elawar M. C. Corno L. (1985). A factorial experiment in teachers’ written feedback on student homework: changing teacher behavior a little rather than a lot. J. Educ. Psychol. 77, 162–173. doi: 10.1037/0022-0663.77.2.162

[ref15] Feng M. Heffernan N. Collins K. Heffernan C. Murphy R. F. (2023). “Implementing and evaluating ASSISTments online math homework support at large scale over two years: findings and lessons learned” in Artificial intelligence in education. eds. Wang N. Rebolledo-Mendez G. Matsuda N. Santos O. C. Dimitrova V. (Switzerland: Springer Nature), 28–40.

[ref16] Figueroa-Cañas J. Sancho-Vinuesa T. (2021). Investigating the relationship between optional quizzes and final exam performance in a fully asynchronous online calculus module. Interact. Learn. Environ. 29, 33–43. doi: 10.1080/10494820.2018.1559864

[ref17] Fyfe E. R. (2016). Providing feedback on computer-based algebra homework in middle-school classrooms. Comput. Human Behav. 63, 568–574. doi: 10.1016/j.chb.2016.05.082

[ref18] Garrison D. R. (2003). E-learning in the 21st century: a framework for research and practice. London: Routledge.

[ref19] Hattie J. Timperley H. (2007). The power of feedback. Rev. Educ. Res. 77, 81–112. doi: 10.3102/003465430298487

[ref20] Hedges L. V. Cooper H. M. Valentine J. C. (2009). The handbook of research synthesis and meta-analysis. 2nd Edn. New York: Russell Sage Foundation.

[ref21] Heffernan N. T. Feng M. (2006). Informing teachers live about student learning: reporting in the assistment system. Technol. Instr. Cogn. Learn. 3. https://web.cs.wpi.edu/Research/trg/public/project/papers/journals/feng_etal.pdf

[ref22] Higgins J. P. T. Thompson S. G. Deeks J. J. Altman D. G. (2003). Measuring inconsistency in meta-analyses. BMJ 327, 557–560. doi: 10.1136/bmj.327.7414.557, 12958120 PMC192859

[ref23] John R. A. Ray P. 1991 A development system for model-tracing tutors. Hillsdale, NJ: Lawrence Erlbaum Associates.

[ref24] Kingston N. Nash B. (2011). Formative assessment: a meta-analysis and a call for research. Educ. Meas. Issues Pract. 30, 28–37. doi: 10.1111/j.1745-3992.2011.00220.x

[ref25] Kodippili A. Senaratne D. (2008). Is computer-generated interactive mathematics homework more effective than traditional instructor-graded homework? Br. J. Educ. Technol. 39, 928–932. doi: 10.1111/j.1467-8535.2007.00794.x

[ref26] Kulik J. A. Kulik C.-L. C. (1988). Timing of feedback and verbal learning. Rev. Educ. Res. 58, 79–97.

[ref27] Leong K. E. Alexander N. (2014). College students attitude andmathematics achievement usingweb based homework. EURASIA J. Math. Sci. Technol. Educ. 10, 609–615. doi: 10.12973/eurasia.2014.1220a

[ref28] Lipsey M. W. Wilson D. B. (2001). Practical meta-analysis (1st Edn, SAGE Publications, Inc. Available online at: https://methods.sagepub.com/book/practical-meta-analysis (Accessed March 15, 2025).

[ref29] Lu Q. Huiyong F. (2020). A meta-analysis of the differences in learning outcomes between online and traditional homework. Open Educ. Res. 26, 100–110.

[ref30] Lucas A. R. (2012). Using *WeBWorK*, a web-based homework delivery and grading system, to help prepare students for active learning. Primus 22, 97–107. doi: 10.1080/10511970.2010.497834

[ref31] Magalhães P. Ferreira D. Cunha J. Rosário P. (2020). Online vs traditional homework: a systematic review on the benefits to students’ performance. Comput. Educ. 152:103869. doi: 10.1016/j.compedu.2020.103869

[ref32] Mendicino M. Razzaq L. Heffernan N. T. (2009). A comparison of traditional homework to computer-supported homework. J. Res. Technol. Educ. 41, 331–359. doi: 10.1080/15391523.2009.10782534

[ref33] Metcalfe J. Kornell N. Finn B. (2009). Delayed versus immediate feedback in children’s and adults’ vocabulary learning. Mem. Cogn. 37, 1077–1087. doi: 10.3758/MC.37.8.1077, 19933453

[ref34] Palocsay S. W. Stevens S. P. (2008). A study of the effectiveness of web-based homework in teaching undergraduate business statistics. Decis. Sci. J. Innov. Educ. 6, 213–232. doi: 10.1111/j.1540-4609.2008.00167.x

[ref35] Paschal R. A. Weinstein T. Walberg H. J. (1984). The effects of homework on learning: a quantitative synthesis. J. Educ. Res. 78, 97–104. doi: 10.1080/00220671.1984.10885581

[ref36] Patall E. A. Cooper H. Robinson J. C. (2008). Parent involvement in homework: a research synthesis. Rev. Educ. Res. 78, 1039–1101. doi: 10.3102/0034654308325185

[ref37] Ramdass D. Zimmerman B. J. (2011). Developing self-regulation skills: the important role of homework. J. Adv. Acad. 22, 194–218. doi: 10.1177/1932202X1102200202

[ref38] Roschelle J. Feng M. Murphy R. F. Mason C. A. (2016). Online mathematics homework increases student achievement. AERA Open 2:2332858416673968. doi: 10.1177/2332858416673968

[ref39] Schubert D. J. (2012). The effects of online homework on high school algebra students. New York: Teachers College, Columbia University.

[ref40] Sergeeva O. V. Masalimova A. R. Zheltukhina M. R. Chikileva L. S. Lutskovskai L. Y. Luzin A. (2025). Impact of digital media literacy on attitude toward generative AI acceptance in higher education. Front. Educ. 10:1563148. doi: 10.3389/feduc.2025.1563148

[ref41] Shute V. J. (2008). Focus on formative feedback. Rev. Educ. Res. 78, 153–189. doi: 10.3102/0034654307313795

[ref42] Singh R. Saleem M. Pradhan P. Heffernan C. Heffernan N. T. Razzaq L. . (2011). “Feedback during web-based homework: the role of hints” in Artificial intelligence in education. eds. Biswas G. Bull S. Kay J. Mitrovic A. (Berlin Heidelberg: Springer), 328–336.

[ref43] Smolira J. C. (2008). Student perceptions of online homework in introductory finance courses. J. Educ. Bus. 84, 90–95. doi: 10.3200/JOEB.84.2.90-95

[ref44] Song F. Gilbody S. (1998). Bias in meta-analysis detected by a simple, graphical test. Increase in studies of publication bias coincided with increasing use of meta-analysis. BMJ 316:471. PMC2665616 9492690

[ref45] Taylor T. M. (2019). The impact of completing homework online versus traditional homework on the attitude, motivation, and academic growth and achievement of 7th grade students in an urban public school [doctoral dissertation]: Trevecca Nazarene University.

[ref46] Tokac U. Novak E. Thompson C. G. (2019). Effects of game-based learning on students’ mathematics achievement: a meta-analysis. J. Comput. Assist. Learn. 35, 407–420. doi: 10.1111/jcal.12347

[ref47] Tran T. N. A. Nguyen Thanh P. (2023). Effects of socrative-based online homework on learning outcomes in Vietnam: a case study. Int. J. Interact. Mobile Technol. 17, 182–199. doi: 10.3991/ijim.v17i05.37513

[ref48] Trautwein U. (2007). The homework–achievement relation reconsidered: differentiating homework time, homework frequency, and homework effort. Learn. Instr. 17, 372–388. doi: 10.1016/j.learninstruc.2007.02.009

[ref49] Vanzo A. Chowdhury S. P. Sachan M. (2024). GPT-4 as a homework tutor can improve student engagement and learning outcomes (no. arXiv:2409.15981). arXiv. doi: 10.48550/arXiv.2409.15981

[ref50] Williams A. (2012). Online homework vs. traditional homework: statistics anxiety and self-efficacy in an educational statistics course. Technol. Innov. Stat. Educ. 6. doi: 10.5070/T561011683

[ref51] Wu D. Li H. Zhu S. Yang H. H. Bai J. Zhao J. . (2024). Primary students’ online homework completion and learning achievement. Interact. Learn. Environ. 32, 4469–4483. doi: 10.1080/10494820.2023.2201343

[ref52] Yang H. H. Kwok L. F. Wang X. (2020). Home-based learning during COVID-19 outbreak: feedback from Chinese parents. Teachers College Record. Available online at: https://www.tcrecord.org/PrintContent.asp?ContentID=23295

[ref53] Zhou X. Wang J. Ma X. (2013). Design and implementation of an anti-plagiarism system for e-homework based on vector space model. Exp. Technol. Manage. 30, 109–111. doi: 10.16791/j.cnki.sjg.2013.03.031

