# Supplementary material for: The effect of E-homework on K-12 students’ academic achievements: a meta-analysis study
Source: Front Psychol. 2026 Jan 30;17:1758739. doi: 10.3389/fpsyg.2026.1758739 (PMC12901446; doi:10.3389/fpsyg.2026.1758739)
Supplement: Supplementary file 1 [file Data_Sheet_1.pdf]

Supplementary Material

1 Supplementary Figures and Tables

1.1 Supplementary Figures

Std diff in means	Subgroup within study	Standard error	Variance	Lower limit	Upper limit	Z-Value	p-Value	Study name
0.610	Mathematics	0.261	0.068	0.099	1.121	2.339	0.019	Michael Mendicino et al. (2009)
0.567	Mathematics	0.256	0.065	0.066	1.068	2.217	0.027	Kim Kelly1 et al. (2014)
0.370	Mathematics	0.160	0.026	0.056	0.684	2.313	0.021	Paul Kehrer et al. (2013)
0.400	Mathematics	0.185	0.034	0.037	0.763	2.158	0.031	Ravi Singh1 et al. (2011)
0.220	Mathematics	0.036	0.001	0.150	0.290	6.163	0.000	Robert Murphy et al. (2020)
0.180	Mathematics	0.418	0.175	-0.640	1.000	0.430	0.667	Jeremy Roschelle et al. (2016)
0.663	Mathematics	0.242	0.059	0.189	1.138	2.739	0.006	Xuetan Zhai a et al. (2023)
0.411	Mathematics	0.135	0.018	0.147	0.676	3.046	0.002	Slavisa?Radovic et al. (2024)
0.251	Language	0.026	0.001	0.201	0.301	9.806	0.000	Alessandro Vanzo Sankalan Pal Chowdhury Mrinmaya Sachan et al. (2024)
0.100	Mathematics	0.026	0.001	0.050	0.150	3.921	0.000	Mingyu Feng et al. (2023)
1.494	Other	0.130	0.017	1.240	1.747	11.533	0.000	Tran Thi Ngoc Anh1 et al. (2023)
0.644	Mathematics	0.223	0.050	0.206	1.082	2.883	0.004	Emily R. Fyfe et al. (2016)
0.270	Mathematics	0.087	0.008	0.100	0.440	3.115	0.002	Mingyu Feng (WestEd) Jeremy Roschelle (Digital Promise) et al. (2016)
0.888	Mathematics	0.327	0.107	0.246	1.530	2.711	0.007	Chad Williams et al. (2022)
0.148	Mathematics	0.366	0.134	-0.568	0.865	0.405	0.685	Jennifer Zakrzewski et al. (2015)
0.875	Language	0.285	0.081	0.316	1.434	3.069	0.002	Hope McGee Nordstrom et al. (2012)
0.558	Mathematics	0.077	0.006	0.408	0.708	7.272	0.000	Daniel Joseph Schubert et al. (2012)
0.220	Mathematics	0.038	0.001	0.146	0.295	5.789	0.000	Jeremy Roschelle et al. (2017)
0.409	Other	0.188	0.035	0.042	0.777	2.181	0.029	Ibrahim Abdullah Ali Alhumaidan et al. (2011)
0.164	Mathematics	0.090	0.008	-0.014	0.341	1.810	0.070	TAMMY S MCCOLLUM et al. (2018)
0.210	Mathematics	0.187	0.035	-0.157	0.577	1.122	0.262	I. Peter Swire et al. (2010)
-1.169	Mathematics	0.253	0.064	-1.666	-0.672	-4.611	0.000	Tanisha Marie Taylor et al. (2019)
-2.008	Language	0.362	0.131	-2.717	-1.299	-5.549	0.000	Pao-Nan Chou et al. (2017)
0.099	Mathematics	0.246	0.061	-0.384	0.582	0.403	0.687	Guo Qi et al. (2018)
0.121	Language	0.258	0.067	-0.385	0.628	0.470	0.639	Xu Fengyi et al. (2021)
0.091	Mathematics	0.195	0.038	-0.291	0.474	0.468	0.640	Lü Jing et al. (2017)
0.223		0.014	0.000	0.197	0.250	16.392	0.000	

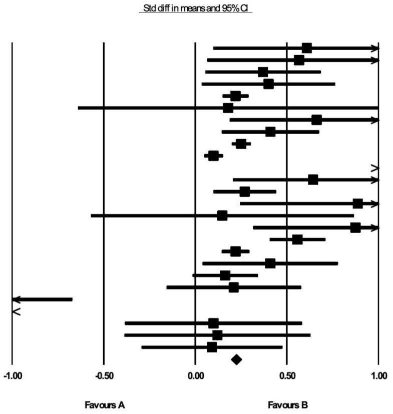

Meta Analysis

Figure S1. Forest plot of subgroup analysis by school level

Std diff in means	Subgroup within study	Standard error	Variance	Lower limit	Upper limit	Z-Value	p-Value	Study name
0.610	Mathematics	0.261	0.068	0.099	1.121	2.339	0.019	Michael Mendicino et al. (2009)
0.567	Mathematics	0.256	0.065	0.066	1.068	2.217	0.027	Kim Kelly1 et al. (2014)
0.370	Mathematics	0.160	0.026	0.056	0.684	2.313	0.021	Paul Kehrer et al. (2013)
0.400	Mathematics	0.185	0.034	0.037	0.763	2.158	0.031	Ravi Singh1 et al. (2011)
0.220	Mathematics	0.036	0.001	0.150	0.290	6.163	0.000	Robert Murphy et al. (2020)
0.180	Mathematics	0.418	0.175	-0.640	1.000	0.430	0.667	Jeremy Roschelle et al. (2016)
0.663	Mathematics	0.242	0.059	0.189	1.138	2.739	0.006	Xuetan Zhai a et al. (2023)
0.411	Mathematics	0.135	0.018	0.147	0.676	3.046	0.002	Slavisa?Radovic et al. (2024)
0.251	Language	0.026	0.001	0.201	0.301	9.806	0.000	Alessandro Vanzo Sankalan Pal Chowdhury Mrinmaya Sachan et al. (2024)
0.100	Mathematics	0.026	0.001	0.050	0.150	3.921	0.000	Mingyu Feng et al. (2023)
1.494	Other	0.130	0.017	1.240	1.747	11.533	0.000	Tran Thi Ngoc Anh1 et al. (2023)
0.644	Mathematics	0.223	0.050	0.206	1.082	2.883	0.004	Emily R. Fyfe et al. (2016)
0.270	Mathematics	0.087	0.008	0.100	0.440	3.115	0.002	Mingyu Feng (WestEd) Jeremy Roschelle (Digital Promise) et al. (2016)
0.888	Mathematics	0.327	0.107	0.246	1.530	2.711	0.007	Chad Williams et al. (2022)
0.148	Mathematics	0.366	0.134	-0.568	0.865	0.405	0.685	Jennifer Zakrzewski et al. (2015)
0.875	Language	0.285	0.081	0.316	1.434	3.069	0.002	Hope McGee Nordstrom et al. (2012)
0.558	Mathematics	0.077	0.006	0.408	0.708	7.272	0.000	Daniel Joseph Schubert et al. (2012)
0.220	Mathematics	0.038	0.001	0.146	0.295	5.789	0.000	Jeremy Roschelle et al. (2017)
0.409	Other	0.188	0.035	0.042	0.777	2.181	0.029	Ibrahim Abdullah Ali Alhumaidan et al. (2011)
0.164	Mathematics	0.090	0.008	-0.014	0.341	1.810	0.070	TAMMY S MCCOLLUM et al. (2018)
0.210	Mathematics	0.187	0.035	-0.157	0.577	1.122	0.262	I. Peter Swire et al. (2010)
-1.169	Mathematics	0.253	0.064	-1.666	-0.672	-4.611	0.000	Tanisha Marie Taylor et al. (2019)
-2.008	Language	0.362	0.131	-2.717	-1.299	-5.549	0.000	Pao-Nan Chou et al. (2017)
0.099	Mathematics	0.246	0.061	-0.384	0.582	0.403	0.687	Guo Qi et al. (2018)
0.121	Language	0.258	0.067	-0.385	0.628	0.470	0.639	Xu Fengyi et al. (2021)
0.091	Mathematics	0.195	0.038	-0.291	0.474	0.468	0.640	Lü Jing et al. (2017)
0.223		0.014	0.000	0.197	0.250	16.392	0.000	

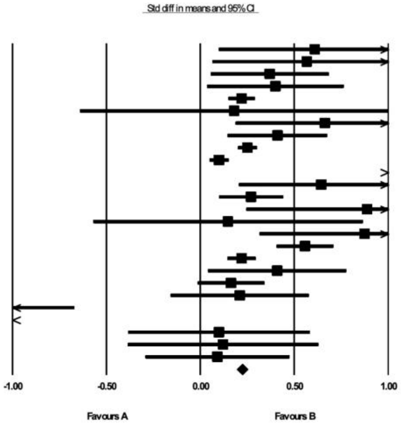

Meta Analysis

Figure S2. Forest plot of subgroup analysis by subject domain

1.2 Supplementary Tables

Table S1. Descriptive Information of Included Original Research Studies

Original Research	Sample Size	Effect Size (Hedges' g)	Educational Stage	Subject Domain	Outcome Type	Publication Type	Feedback Type	Intervention Duration
Mendicino et al., 2009	28	0.5922	ES	Math	AP	J	Ins	ST
Kelly et al., 2014	63	0.56	MS	Math	AP	C	Bas	ST
Kehrer et al., 2013	61	0.365	MS	Math	AP	J	Ins	ST
Singh et al., 2011	68	0.396	MS	Math	AP	C	Ins	ST
Murphy et al., 2020	2769	0.22	MS	Math	EP	J	Ins	MT
Roschelle et al., 2016	2850	0.18	MS	Math	EP	J	Ins	LT
Zhai et al., 2023	81	0.656	ES	Math	EP	J	Ins	ST
Radović, 2024	325	0.41	ES	Math	EP	J	Ins	MT
Vanzo et al., 2024	76	0.248	HS	Lang	EP	C	Ins	Oth
Feng et al., 2023	5991	0.1	MS	Math	EP	J	Ins	LT
Tran et al., 2023	305	1.49	HS	Other	EP	J	Bas	ST

Fyfe, 2016	143	0.641	MS	Math	AP	J	Ins	Oth
Feng et al., 2016	1555	0.27	MS	Math	AP	J	Bas	LT
Williams, 2022	13	0.831	MS	Math	AP	T	Bas	MT
Zakrzewski, 2015	30	0.144	ES	Math	AP	J	Ins	MT
McGee-Nordstrom, 2012	54	0.862	ES	Lang	EP	T	Bas	MT
Schubert, 2012	95	0.554	MS	Math	EP	T	Ins	ST
Roschelle et al., 2017	Unreported	0.22	MS	Math	EP	J	Ins	LT
Alhumaidan, 2011	116	0.41	MS	Lang	AP	J	Ins	ST
McCollum, 2018	124	0.163	ES	Math	EP	T	Bas	MT
Swire et al., 2010	131	0.209	MS	Math	EP	J	Ins	ST
Taylor et al., 2019	73	-1.156	MS	Math	EP	T	Bas	ST
Chou et al., 2017	46	-1.974	MS	Lang	EP	J	Ins	ST
Guo, 2018	105	0.098	ES	Math	AP	T	Ins	ST
Xu, 2021	90	0.120	MS	Lang	EP	T	Ins	LT

Lv, 2017	68	0.091	ES	Math	EP	T	Ins	ST
